# Role of brain‐derived neurotrophic factor in endotoxaemia‐induced acute lung injury

**DOI:** 10.1113/EP091228

**Published:** 2023-11-01

**Authors:** Jinye Shi, Shuang Song, Kaixuan Wu, Gui Liang, Aizhong Wang, Xiaotao Xu

**Affiliations:** ^1^ College of Fisheries and Life Science Shanghai Ocean University Shanghai China; ^2^ Department of Anesthesiology, Affiliated Shanghai Sixth People's Hospital Shanghai Jiao Tong University Shanghai China; ^3^ Department of Respiratory Medicine, Affiliated Shanghai Sixth People's Hospital Shanghai Jiao Tong University Shanghai China

**Keywords:** acute lung injury, autophagy, brain‐derived neurotrophic factor

## Abstract

Acute lung injury (ALI) or acute respiratory distress syndrome (ARDS), which is a pulmonary manifestation of a systemic reactive inflammatory syndrome, is a serious disease with high mortality, and sepsis is an important risk factor in the development of ALI. Brain‐derived neurotrophic factor (BDNF) is a member of the nerve growth factor family. It plays an essential role in the regulation of the modification of synaptic efficacy and brain metabolic activity and enhances neuronal survival. However, the role and underlying mechanism of BDNF in sepsis‐induced ALI remain unclear. Here, we sought to observe the expression of BDNF in the lung tissues of mice. C57BL/6J mice were divided randomly into two groups: saline (*n* = 4) and lipopolysaccharide (LPS) (*n* = 4). We found that BDNF expression was elevated in the lung tissues of septic mice. Furthermore, we found that BDNF colocalized with aquaporin 5, a marker for type I alveolar epithelial cells, by immunofluorescence staining. In addition, we also found that tropomyosin‐related kinase B, the specific receptor of BDNF, colocalized with surfactant protein C, a marker for type II alveolar epithelial cells, by immunofluorescence staining. Finally, the present study indicated that BDNF may alleviate excessive LPS‐induced autophagy in alveolar epithelial cells. Overall, we hypothesize that BDNF expression increases in the lung tissues of septic mice as a compensatory mechanism to ameliorate sepsis‐induced ALI by inhibiting excessive alveolar epithelial cell autophagy.

## Abstract (highlights)

- **What is the central question of this study?**
  Does brain‐derived neurotrophic factor (BDNF) expressed in type I alveolar epithelial cells exert a protective effect in sepsis‐induced acute lung injury (ALI)?
- **What is the main finding and its importance?**
  BDNF was mainly coexpressed with aquaporin 5 (expressed in type I alveolar epithelial cells). In contrast, BDNF was less colocalized with F4/80 (expressed in alveolar macrophages) or surfactant protein C (expressed in type II alveolar epithelial cells). The increased expression of BDNF protein in the lung tissues of septic mice as a compensatory mechanism may alleviate sepsis‐induced ALI by inhibiting excessive cellular autophagy.

## 1 INTRODUCTION

Sepsis is a life‐threatening organ dysfunction syndrome that afflicts millions of patients worldwide each year. The mortality rate of these patients is 30–35% without effective and timely intervention (Singer et al., 2016; Vincent et al., 2019). Septicaemia is the leading cause of acute lung injury (ALI)/acute respiratory distress syndrome (ARDS) (Butt et al., 2016; Meyer et al., 2021). A growing body of literature has demonstrated that sepsis could enhance the production of inflammatory mediators and reactive oxygen species and exacerbate lung tissue damage (Lelubre & Vincent, 2018). Therefore, strategies to suppress inflammatory and oxidative stress in the sepsis setting are critical to preserve cellular homeostasis and may result in a better prognosis.

Brain‐derived neurotrophic factor (BDNF) is a specific class of factors widely distributed in the brain that play an important role in depression and neurodegenerative diseases. BDNF can act by binding to the specific receptor tropomyosin‐related kinase B (TrkB), a class of cell membrane surface receptors, the absence of which has been shown to cause rapid death in mice (Prakash et al., 2010). BDNF can also be used as a biomarker of treatment response in patients with treatment‐resistant depression (Meshkat et al., 2022). In addition to neurological‐related diseases (Bjorkholm & Monteggia, 2016; Marosi & Mattson, 2014), BDNF is also associated with non‐neuronal diseases, including those of the liver (Girard et al., 2021), muscle (Pedersen & Febbraio, 2012), lung (Ray et al., 2022), etc. BDNF plays a major role in neurogenesis, neurite outgrowth, connectivity, differentiation, neuronal migration (Bhattarai et al., 2020; Zhang et al., 2021) and synapse formation (Leal et al., 2014). BDNF has been shown to be crucial for axonal transport (Du et al., 2020) and a self‐amplifying positive feedback mechanism mediated by cyclic AMP, protein kinase A and phosphatidylinositol 3‐kinase (PI3 kinase) pathways to promote BDNF secretion, trigger TrkB membrane insertion and stimulate anterograde axonal transport, thereby leading to the formation of neuronal polarity and axonal development (Cheng et al., 2011).

Additionally, to some extent, BDNF has an anti‐inflammatory effect (Dong et al., 2018; Zhang et al., 2016). Research on BDNF has mostly focused on neurodegenerative diseases or depression, while only a few researchers have linked it to lung disease (Ray et al., 2022). In fact, the role of BDNF in the lungs should not be underestimated, and its presence is closely related to lung development and health (Prakash et al., 2010). One study found that BDNF‐positive cell subtypes play a vital role in promoting early epithelial development during lung development by secreting abundant growth factors, which contribute to the formation of intricate epithelial branch morphology (Cao et al., 2023). Upregulation of BDNF expression can be found in bronchopulmonary dysplasia, rhinitis, pulmonary fibrosis and other diseases (Prakash et al., 2010). In addition, it has been reported that BDNF plays a major role in acid‐induced lung injury (Paris et al., 2020). However, the role and underlying mechanism of BDNF in endotoxaemia‐induced ALI remains unexplored. Accordingly, we investigated the expression and role of BDNF in ALI induced by sepsis in mice. We sought to test the hypothesis that exogenous BDNF or the TrkB agonist 7,8‐dihydroxyflavone (7,8‐DHF) alleviates endotoxaemia‐induced ALI by inhibiting excessive cellular autophagy.

## 2 METHODS

### 2.1 Ethical approval

All animal experiments and procedures were conducted in accordance with principles set out by Grundy (2015) and the ARRIVE guidelines and were approved by the Institutional Animal Care and Use Committee of Sixth People's Hospital affiliated with Shanghai Jiao Tong University (permit number: 2022‐0486).

### 2.2 Animals

Male C57BL/6 mice (7 weeks of age) used in the experiments were purchased from Shanghai JSJ Laboratory Animal Company and housed in the animal house of the Sixth People's Hospital of Shanghai Jiaotong University. The environment consisted of a 12 h light cycle to simulate the normal circadian rhythm, specific pathogen‐free rooms with a temperature of 20–26°C and 50–60% humidity and a standard rodent diet and water provided ad libitum. After an initial acclimation period, mice (8 weeks of age, weighing approximately 23 g) were randomly divided into two groups: a control group with intraperitoneal injection of the same volume of saline, and a lipopolysaccharide (LPS) group with intraperitoneal injection of 10 mg/kg LPS. After 24 h, mice were anaesthetized via a single intraperitoneal injection of 4% pentobarbital (40 mg/kg). The level of anaesthesia was assessed regularly by evaluating limb withdrawal reflexes in response to noxious pinching, with a wait time of approximately 2–3 min. And then thoracotomy was performed once the animal reached the desired level of the ‘deep surgical stage’. The right ventricle was intubated into the pulmonary artery, and the left auricle of the heart was cut to drain the perfusate. Saline was first perfused until the lung tissues became pale and the left atrial outflow was clear. At the end of perfusion using saline, we excised the right lung for subsequent western blot experiments. Subsequently, the left lung was perfused using 20 mL of 4% paraformaldehyde for fixation. After completing the perfusion process, the left lung was excised and then immersed in 5 mL of 4% paraformaldehyde for subsequent paraffin embedding. At the end of sample collection, mice were killed by exsanguination.

### 2.3 Cell culture and treatment

The cells (MLE‐12) used for the experiment were purchased from the Cell Bank of the Chinese Academy of Sciences (Shanghai, China). Cells were cultured in Dulbecco's modified Eagle's medium (cat. no. SH30243.01, Cytiva, Marlborough, MA, USA; 4.5 g/mL glucose) supplemented with 10% fetal bovine serum (cat. no. 10091e148, Thermo Fisher Scientific, Waltham, MA, USA) at 37°C in a 5% CO_2_ incubator. Cells were pretreated with 16 µM or 32 µM BDNF mimics (7,8‐DHF) and then treated with 10 mg/L LPS for 24 h.

### 2.4 Determination of cell viability

To assess cell viability, MLE‐12 cells were seeded in 96‐well plates at a density of 10^4^ cells/well and treated with 7,8‐DHF (1‐32 µM) for 1 h prior to exposure to LPS (10 mg/L) for 24 h. Diluted Cell Counting Kit‐8 (CCK‐8) (10 µL dissolved in 100 µL of basal medium) was added to the cells and incubated for 2 h at 37°C before measuring absorbance at 450 nm using an enzyme labelling instrument (Molecular Devices, San Jose, CA, USA). Viability was calculated as a percentage of untreated control cells.

### 2.5 Histological analysis

Mouse lung tissues were examined in histological sections as described previously (Xu et al., 2018). Briefly, after mice were anaesthetized, lung tissues were perfused with saline, and then left lung tissue was collected and fixed in 4% paraformaldehyde (PFA). After fixation, the tissue samples were embedded in paraffin and sectioned. Staining was performed using haematoxylin and eosin (H&E). Histopathological changes in lung tissue were observed under an inverted microscope.

### 2.6 Western blot analysis

After mice were anaesthetized, lung tissues were quickly perfused, and right lung tissue was harvested and then stored in a −80°C freezer for western blot analysis. Equal amounts of protein (15–30 µg) were separated by 12–15% SDS‐PAGE, and then transferred to polyvinylidene fluoride membranes. The membranes were blocked with phosphate‐buffered saline (PBS) containing 5% w/v non‐fat milk for 2 h and then incubated with the primary antibodies. BDNF was detected by Huabio (Hangzhou, China) antibodies (1:1000). SQSTM1/P62 was detected by Abcam (Cambridge, UK) antibodies (1:30000). LC3A/B was detected by a Cell Signaling Technology (Danvers, MA, USA) antibody (1:1000). β‐Actin was detected by a Cell Signaling Technology antibody (1:5000). After washing with Tris‐buffered saline containing 0.1% v/v Tween 20, the membranes were then incubated with a horseradish peroxidase‐conjugated secondary antibody for 1 h at ambient temperature and developed using an enhanced chemiluminescence detection kit (MilliporeSigma, Burlington, MA, USA). Images were acquired using ImageQuant LAS 4000 mini (GE Healthcare Life Sciences, Chicago, IL, USA).

### 2.7 Immunofluorescence staining

Immunofluorescence staining was carried out as described previously (Xu et al., 2021). After mice were anaesthetized, lung tissues were perfused with saline, and then left lung tissue was collected and fixed in 4% PFA. After fixation, the tissue samples were embedded in paraffin, sectioned and dewaxed. Tissue sections were blocked in 5% goat serum, 0.4% Triton X‐100 and 3% bovine serum albumin in 1× PBS for 2 h at room temperature. We applied the mouse anti‐aquaporin 5 (AQP5) antibody (1:200, Santa Cruz Biotechnology, Dallas, TX, USA), mouse anti‐F4/80 antibody (1:5000, Thermo Fisher Scientific), mouse anti‐surfactant protein C (SP‐C) antibody (1:200, Santa Cruz Biotechnology), rabbit anti‐TrkB antibody (1:50, ABclonal Technology, Woburn, MA, USA) or rabbit anti‐BDNF antibody (1:200, Huabio) overnight at 4°C. After washing in 1× PBS three times, the slices were incubated with Alexa Fluor 488‐conjugated anti‐mouse (1:400; Abcam), Alexa Fluor 488‐conjugated anti‐rabbit (1:400; Abcam), Alexa Fluor 594‐conjugated anti‐mouse (1:400; Abcam), and Alexa Fluor 594‐conjugated anti‐rabbit (1:400; Abcam) antibodies for 1 h. Nuclei were stained with 4′,6‐diamidino‐2‐phenylindole (DAPI; Beyotime Institute of Biotechnology, Haimen, China) for 5 min at room temperature. Confocal images were taken using a Leica SP8 X system (Leica Microsystems, Wetzlar, Germany).

### 2.8 Statistical analysis

All data are presented as the mean ± SD and were analysed using GraphPad Prism 9 (GraphPad Software, Boston, MA, USA). Student's unpaired *t‐*test was used for comparing two groups, while one‐way ANOVA followed by Tukey's post‐hoc test was employed for comparing multiple groups (>2 groups). *P* < 0.05 was considered statistically significant.

## 3 RESULTS

### 3.1 BDNF protein expression increased in the lung tissues of septic mice

**FIGURE 1:**
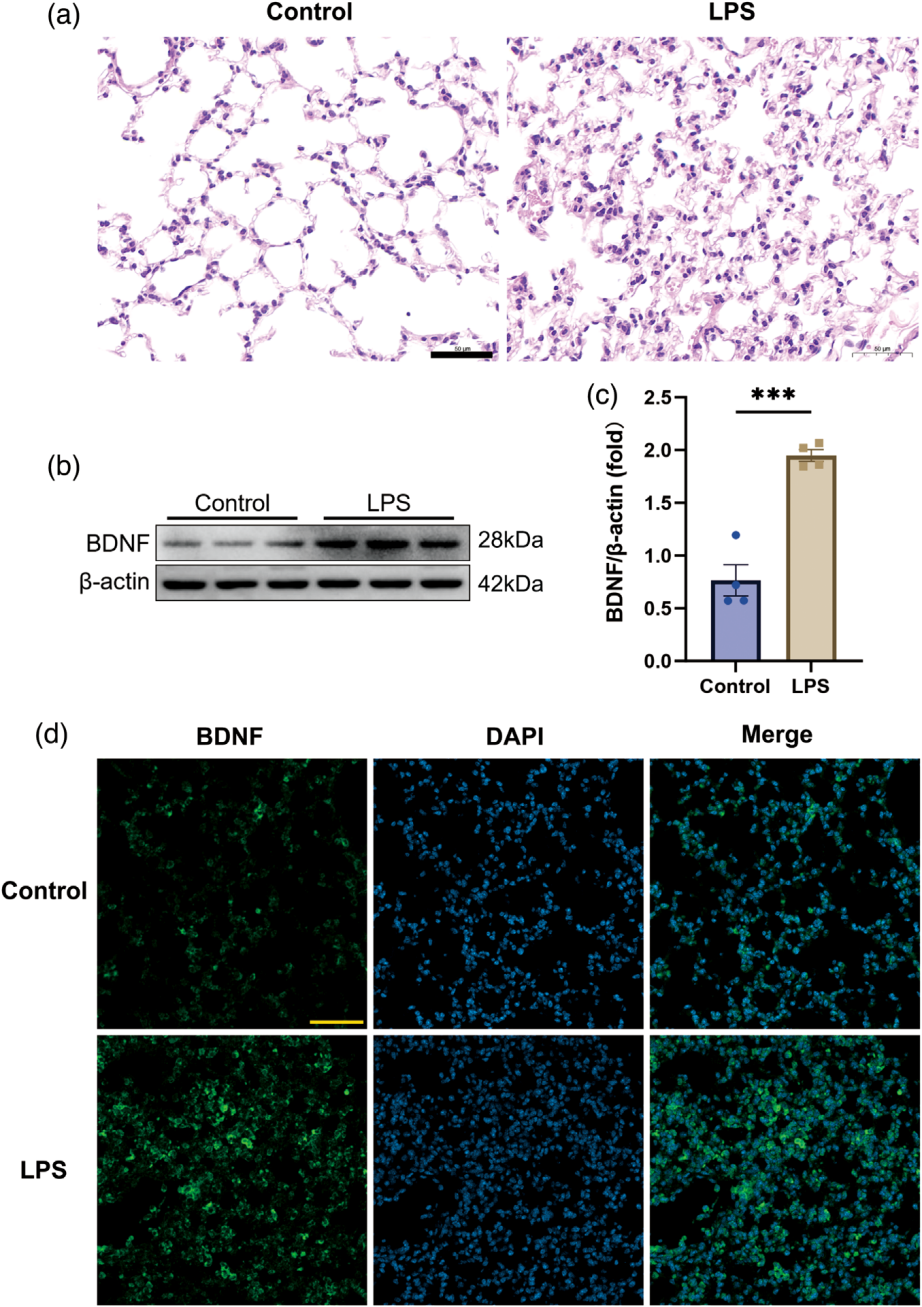
The expression of BDNF increased in the lung tissues of septic mice. (a) H&E staining was used to assess lung injury (*n* = 4, scale bars: 50 µm). (b) The expression of BDNF in lung tissues was determined by western blot analysis (*n* = 4, *P* = 0.0003). (c) Densitometric analysis of the proteins from (b) was performed with normalization to the respective loading control. Scattered circles (control, *n* = 4) and squares (LPS, *n* = 4) represent individual values, and bars represent mean values with the respective SD for each group. Comparisons between groups were performed by Student's unpaired *t*‐test. (d) BDNF (green) and DAPI (blue) double staining in the lung tissues was detected by immunofluorescence (scale bars: 50 µm).

First, to establish a sepsis‐induced ALI animal model, mice were injected with LPS (10 mg/kg, intraperitoneally (i.p.)). After 24 h, the mice were anaesthetized, and lung tissues were collected for H&E staining. Figure 1a shows that alveolar structures were damaged and accompanied by massive inflammatory cell infiltration and alveolar wall thickening in septic mice. We then examined the changes in BDNF expression in lung tissues using western blotting and immunofluorescence staining. Western blotting results showed that the expression of BDNF was significantly elevated in the lung tissues of septic mice versus control animals (Figure 1b, c, *P* = 0.0003). Consistent with the western blotting results, the immunofluorescence staining results also illustrated that the fluorescence intensity of BDNF was significantly greater in the lung tissues of septic mice than in those of control animals (Figure 1d). These data demonstrated that BDNF expression is elevated in the lungs of septic mice (*P* < 0.0001).

### 3.2 BDNF is primarily expressed in alveolar epithelial type I cells in septic mice

**FIGURE 2:**
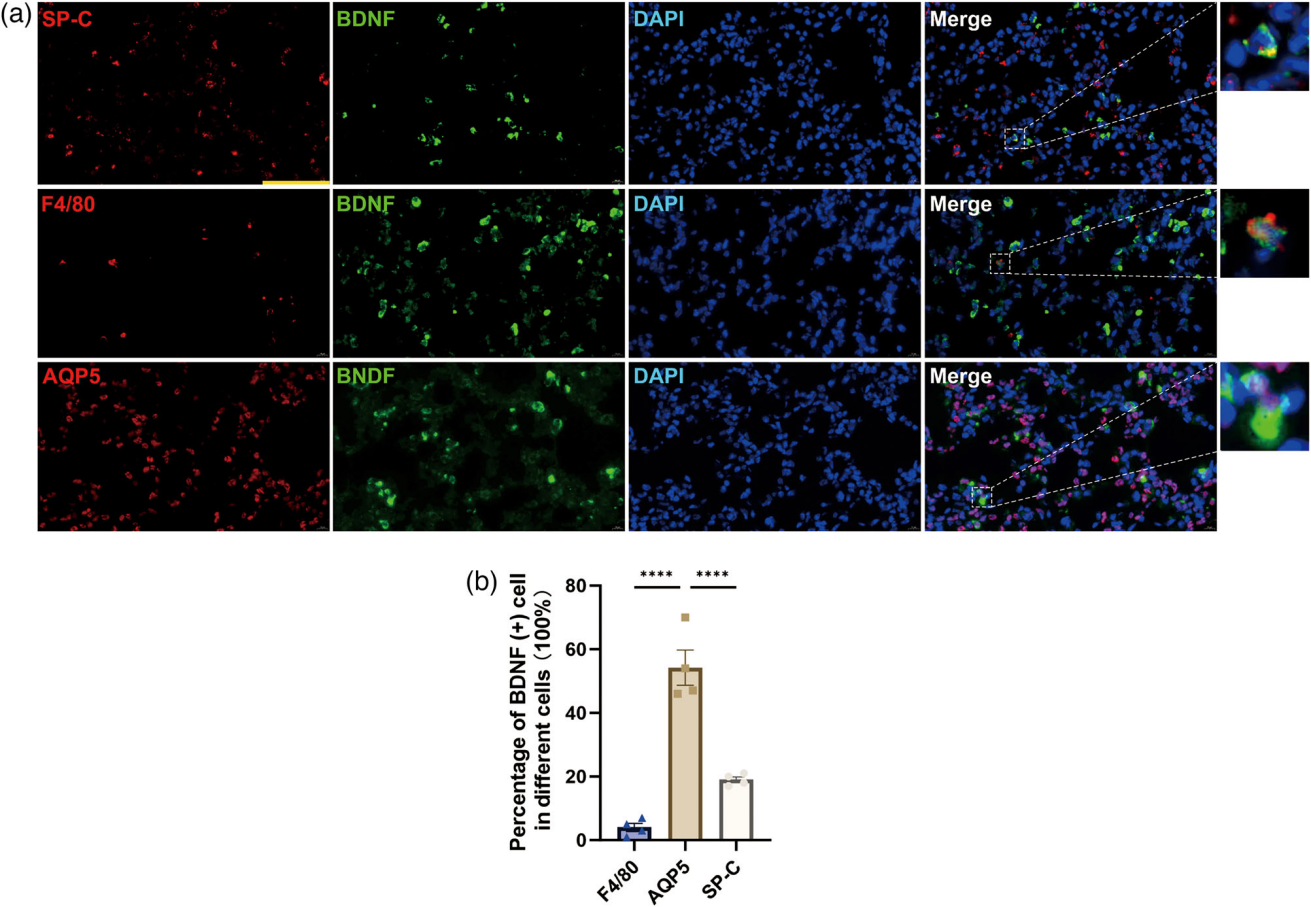
BDNF is primarily colocalized with alveolar epithelial type I cells in septic mice. (a) BDNF (green), DAPI (blue), SP‐C (red; marker of type II alveolar epithelial cells), F4/80 (red; marker of macrophages) and AQP5 (red; marker of type I alveolar epithelial cells) staining in the lung tissues was detected by immunofluorescence (scale bars: 50 µm). (b) BDNF colocalization with different cell types was quantified. Scattered triangles (F4/80, *n* = 4), squares (AQP5, *n* = 4) and circles (SP‐C, *n* = 4) represent individual values, and bars represent mean values with the respective SD for each group. One‐way ANOVA for repeated measurements followed by Tukey's post‐hoc test was used to compare multiple independent groups. ^****^ *P* ≤ 0.0001, AQP5 versus other groups.

To investigate the cell types in which BDNF is mainly expressed in mouse lung tissue, we examined the expression of BDNF using immunofluorescence staining with specific cell markers. As shown in Figure 2a, b, we found that in septic mouse lungs, BDNF was mainly coexpressed with aquaporin 5 (AQP5), a marker for alveolar epithelial type I cells. In contrast, BDNF was less colocalized with F4/80, a marker for alveolar macrophages, or surfactant protein C (SP‐C), a marker for alveolar epithelial type II cells, suggesting that BDNF is mainly expressed in alveolar epithelial type I cells. These results suggested that BDNF protein expression in alveolar epithelial type I cells increased in septic mice.

### 3.3 Type II alveolar epithelial cells were the target cells for BDNF action, and TrkB expression was downregulated

**FIGURE 3:**
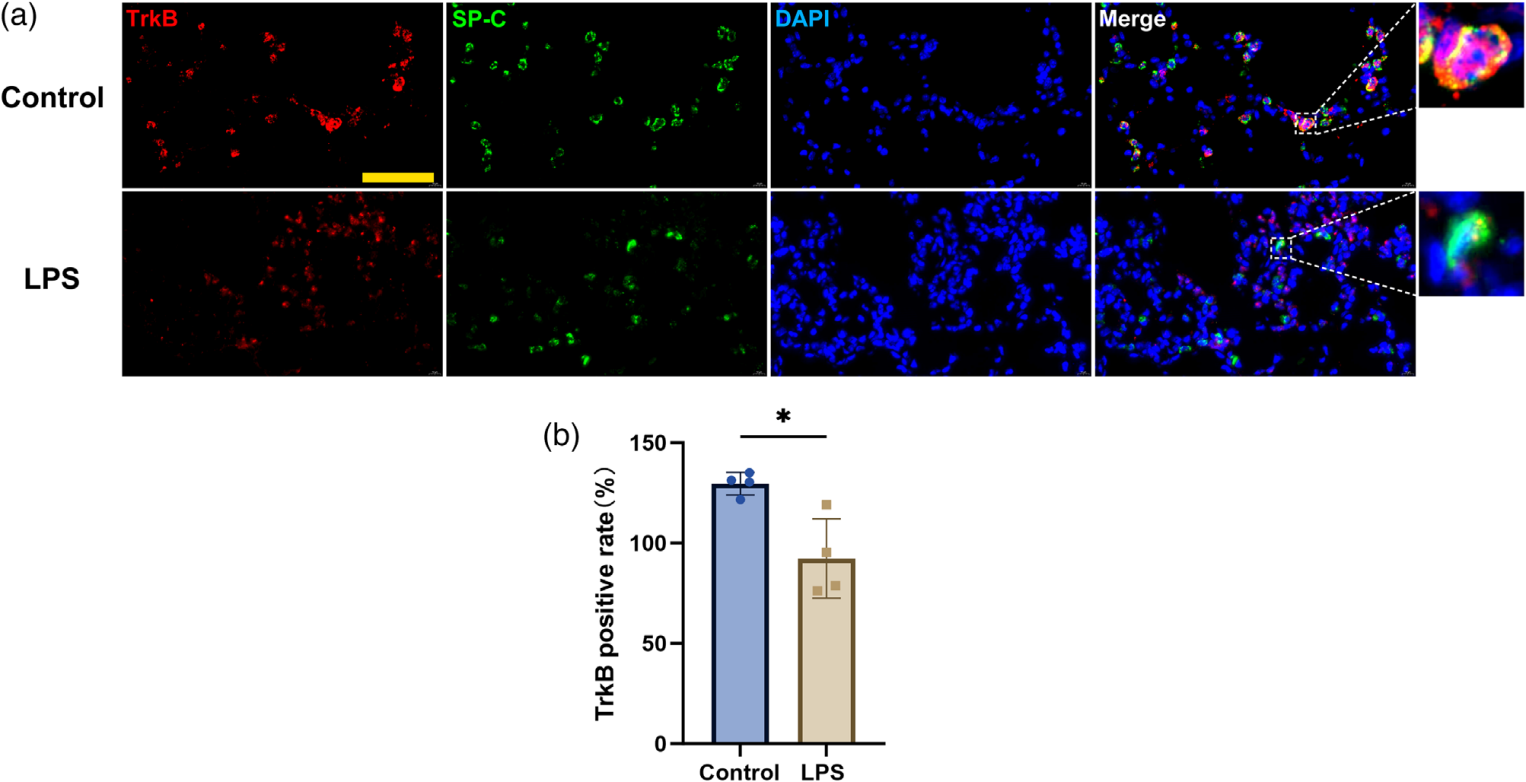
The expression of TrkB in alveolar epithelial type II cells decreased in septic mice. (a) TrkB (red), SP‐C (green) and DAPI (blue) staining in the lung tissues was detected by immunofluorescence (scale bars: 50 µm). (b) Quantification of TrkB was performed in the two groups (*P* = 0.0109). Scattered circles (Control, *n* = 4) and squares (LPS, *n* = 4) represent individual values, and bars represent mean values with the respective SD for each group. Comparisons between groups were performed by Student's unpaired *t*‐test.

Given that the specific receptor of BDNF is TrkB, we also used the immunofluorescence colocalization method to find the cell types expressing TrkB (the target cells of BDNF's actions). We found that TrkB was mainly coexpressed with SP‐C, a marker for alveolar epithelial type II cells, indicating that alveolar epithelial type II cells expressed TrkB and are the target cells of BDNF's action. After detecting the cell type expressing TrkB, we focused on the expression of TrkB protein. Strikingly, TrkB expression was negatively correlated with BDNF expression in the lung tissues of septic mice (Figure 3, *P* = 0.0109). These data suggested that BDNF protein expression in alveolar epithelial type I cells increased and TrkB protein expression in alveolar epithelial type II cells decreased in septic mice.

### 3.4 BDNF mimics alleviate LPS‐induced excessive cellular autophagy

**FIGURE 4:**
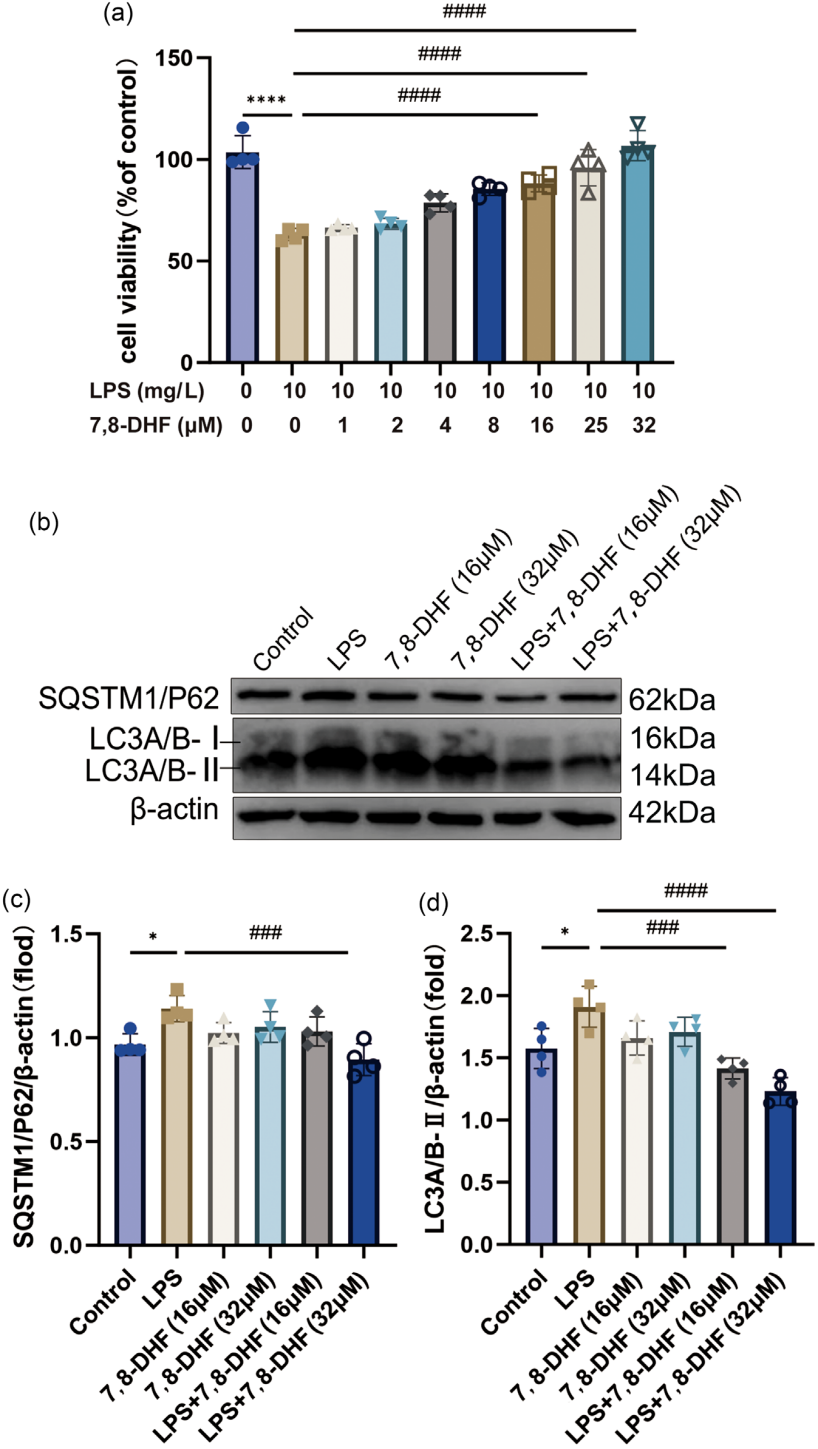
BDNF inhibits excessive LPS‐induced cellular autophagy in MLE‐12 cells. (a) The cells were pretreated with 7,8‐DHF (1–32 µM) for 1 h, then exposed to LPS (10 mg/L) for 24 h. The viability of MLE‐12 cells was determined by the CCK‐8 assay (*n* = 4, ^####^ *P* < 0.0001 LPS vs. other groups). (b) The expression of SQSTM1/P62 (*n* = 4, ^*^ *P* = 0.0154 Control vs. LPS; ^###^ *P* = 0.0006 LPS vs. LPS+7,8‐DHF (32 µM)) and LC3A/B (*n* = 4, ^*^ *P* = 0.0216 Control vs. LPS; ^###^ *P* < 0.0006, ^####^ *P* < 0.0001 LPS vs. other groups) in MLE‐12 cells was determined by western blot analysis. (c, d) Densitometric analysis of the proteins from (b) was performed with normalization to the respective loading control. The results are reported as the mean ± SD of multiple independent experiments. One‐way ANOVA for repeated measurements followed by Tukey's post‐hoc test was used to compare multiple independent groups.

To investigate the function of BDNF in sepsis‐induced ALI in vitro, the alveolar epithelial cell line MLE‐12 was treated with the BDNF mimic 7,8‐dihydroxyflavone (7,8‐DHF) (Ahuja et al., 2022; Bollen et al., 2013; Liu et al., 2016; Xue et al., 2021) (16 µM or 32 µM) for 1 h and then incubated with or without LPS (10 mg/L) for 24 h. The concentration at which 7,8‐DHF was added was selected from previous experimental data (Han et al., 2014) and our experiments (Figure 4a). As shown in Figure 4b–d, the expression of SQSTM1/P62 (*P* = 0.0154) and LC3A/B (*P* = 0.0216), markers for autophagy, increased in LPS‐treated MLE‐12 cells, implying that LPS can cause excessive autophagy in epithelial cells. Moreover, 7,8‐DHF (32 µM) inhibited the LPS‐induced increase in SQSTM1/P62 (*P* = 0.0006) and LC3A/B (*P* < 0.0001) expression. These results demonstrated that BDNF may alleviate LPS‐induced excessive autophagy in epithelial cells. However, the role of BDNF in sepsis‐induced ALI needs to be explored further.

## 4 DISCUSSION

In the present study, we found significant upregulation of BDNF expression in alveolar epithelial type I cells of septic mice. Furthermore, we demonstrated that the BDNF mimic 7,8‐DHF inhibited excessive autophagy in epithelial cells induced by LPS.

There is growing evidence that BDNF can be synthesized and secreted from specific cells at the synapse, including astrocytes (Parpura & Zorec, 2010), microglia (Parkhurst et al., 2013) and postsynaptic dendritic cells (Lessmann & Brigadski, 2009). BDNF is one of the most distributed and well‐characterized neurotrophins in the mammalian central nervous system. BDNF signals through the low affinity p75 neurotrophin receptor (p75NTR) and tropomycin receptor kinase B (TrkB), a member of the larger family of Trk receptors (Colucci‐D'Amato et al., 2020). Many distinct cellular processes involved in the maintenance of normal brain function and development are regulated by BDNF by binding and activating TrkB. In the brain, BDNF is expressed by microglia (Parkhurst et al., 2013), glutamatergic neurons (Andreska et al., 2014) and glial cells, such as astrocytes isolated from the hippocampus and cortex but not from the striatum (Clarke et al., 2018). In addition to the nervous system, BDNF is also detected in several non‐neuronal tissues, such as megakaryocytes (Pius‐Sadowska & Machalinski, 2017), platelets (Chacon‐Fernandez et al., 2016), vascular smooth muscle cells (Nakahashi et al., 2000), leukocytes (Anders et al., 2020), cardiomyocytes (Pius‐Sadowska & Machalinski, 2017) and endothelial cells (Nakahashi et al., 2000). However, whether BDNF is expressed in the lung and which cell types (macrophage, endothelial cells, alveolar epithelial type I cells, alveolar epithelial type II cells, neutrophil cells) express BDNF have not been fully clarified. Here, we found that the expression of BDNF increased in the lung tissues of septic mice. Importantly, we demonstrated that BDNF is mainly expressed by alveolar epithelial type I cells.

BDNF plays an essential role in plasticity, development and proper growth of GABAergic and glutamatergic synapses. Indeed, BDNF influences dopaminergic and serotonergic neurotransmission by modulating neuronal differentiation. BDNF acts as an autocrine and paracrine factor on both presynaptic and postsynaptic target sites (Colucci‐D'Amato et al., 2020). The BDNF–TrkB signaling pathway contributes to the differentiation of cortical progenitor cells and later contributes to the differentiation of cortical progenitor cells into neurons (Bartkowska et al., 2007). It has been reported that the BDNF–TrkB signaling pathway is implicated in adult neurogenesis in the hippocampus and that BDNF–TrkB signaling has distinct roles in the subventricular zone and dentate gyrus of the hippocampus (Vilar & Mira, 2016). Several lines of evidence also suggest that voluntary physical exercise increases BDNF protein expression in the hippocampus (Loprinzi et al., 2019) as well as hippocampal neurogenesis (Fabel et al., 2009). Physical exercise is used as an effective strategy for increasing circulating levels of BDNF and improving brain function (van Praag et al., 2005). Converging data now suggest that deficits in BDNF lead to the pathogenesis of several diseases, such as depression, bipolar disorder, anxiety disorders, Huntington's disease, schizophrenia and Alzheimer's disease. Therefore, regulating BDNF signalling could treat a variety of psychiatric and neurological disorders. Given that the effects of BDNF are mediated by TrkB, we wanted to explore whether TrkB is expressed in the lung and which cell types express TrkB. Therefore, we also performed cellular colocalization of TrkB to determine the true cell type of BDNF action, and immunofluorescence showed that TrkB colocalized with SP‐C (specifically expressed in type II alveolar epithelial cells), suggesting that type II alveolar epithelial cells may be the true target cells of BDNF action. However, in the process of exploring whether TrkB appears to be differentially expressed, we expected that the expression of TrkB would be consistent with the expression trend of its ligand BDNF, but to our surprise, TrkB instead appeared to be downregulated in the lung tissues of septic mice.

To investigate the role of the BDNF pathway, MLE‐12 cells were treated with exogenous BDNF or the TrkB agonist 7,8‐DHF. 7,8‐DHF has been developed for the treatment of neurodegenerative diseases and injuries, including Alzheimer's disease, Huntington's disease, Parkinson's disease, stroke and traumatic brain injury (Agrawal et al., 2015; Castello et al., 2014; Jang et al., 2010; Korkmaz et al., 2014; Wang et al., 2014; Zhang et al., 2014). 7,8‐DHF can bind and activate TrkB in a similar manner to BDNF. However, in addition to its longer half‐life, 7,8‐DHF does not initiate TrkB degradation (Jang et al., 2010; Wurzelmann et al., 2017; Zhang et al., 2014). In the present study, MLE‐12 cells were treated with 7,8‐DHF for 1 h and then incubated with or without LPS (10 mg/L) for 24 h. We found that LPS increased the expression of autophagy‐associated proteins, which was reversed by 7,8‐DHF.

### 4.1 Study limitations

There are several limitations of this study. First, in this study, we only observed that the TrkB agonist 7,8‐DHF can ameliorate the increased expression of autophagy‐associated proteins induced by LPS. However, we did not study whether exogenous administration of BDNF can inhibit the LPS‐induced increase in the expression of autophagy‐associated proteins. 7,8‐DHF has been recognized as a promising small‐molecule BDNF mimetic compound that mimics the physiological actions of BDNF by directly binding to the extracellular domain of TrkB to trigger TrkB receptor dimerization and anti‐phosphorylation (Jang et al., 2010). However, the 7,8‐DHF used in the present study did not completely exclude potential non‐BDNF effects. Here, it would be inappropriate to fully equate the role of 7,8‐DHF with that of BDNF. In addition, selective deletion of BDNF in the alveolar epithelial type I cells of mice and its effect on sepsis‐induced ALI need to be explored in further studies. Second, to avoid bias from animals, a large number of samples are needed to increase reproducibility. Finally, questions remain as to whether there is a sex‐dependent difference in the role of BDNF in sepsis‐induced ALI.

### 4.2 Conclusions

In summary, our novel observations were the following: (1) the expression of BDNF in alveolar epithelial type I cells was upregulated, and the expression of TrkB in alveolar epithelial type II cells was downregulated in septic mice; and (2) the TrkB agonist 7,8‐DHF suppressed the increased expression of autophagy‐associated proteins in LPS‐treated MLE‐12 cells. These findings signal the need for future investigations to explore the role of BDNF in sepsis‐induced ALI and to identify novel strategies that activate BDNF–TrkB signalling to potentially mitigate sepsis‐induced ALI.

## AUTHOR CONTRIBUTIONS

Xiaotao Xu and Aizhong Wang conceived this study. Jinye Shi and Shuang Song performed the experiments. Kaixuan Wu analysed the data. Xiaotao Xu drafted the manuscript. Gui Liang critically reviewed the manuscript. All authors have read and approved the final version of this manuscript and agree to be accountable for all aspects of the work in ensuring that questions related to the accuracy or integrity of any part of the work are appropriately investigated and resolved. All persons designated as authors qualify for authorship, and all those who qualify for authorship are listed.

## CONFLICT OF INTEREST

None declared.

## Data Availability

The data that support the findings of the present study are available from the corresponding author upon reasonable request.
